# Role of Radiofrequency (Votiva, InMode) in Pelvic Floor Restoration

**DOI:** 10.1097/GOX.0000000000002203

**Published:** 2019-04-25

**Authors:** Erez Dayan, Henry Ramirez, Lacy Westfall, Spero Theodorou

**Affiliations:** From the * Massachusetts General Hospital, Harvard Medical School, Boston, Mass.; † Southern Oklahoma Women’s Health, Ardmore, Okla.; ‡ Manhattan Eye, Ear, Throat Hospital, New York, N.Y.

## Abstract

**Background:**

Postpartum pelvic floor disorders are estimated to impact 24% of women in the United States. This study describes the use of a radiofrequency device (Votiva, InMode) for postpartum pelvic floor restoration using an electrostimulator to objectively measure treatment effect.

**Methods:**

A retrospective evaluation was conducted between April 2017 and May 2018 of consecutive patients undergoing vaginal radiofrequency treatment. Inclusion criteria were patients at least 6 weeks postvaginal delivery with symptoms of pelvic floor dysfunction. Resting pelvic floor muscle tone and maximal pelvic floor contraction were measured.

**Results:**

Fifty women were included in the study with an average age of 32 (29–40) years old, average of 2.6 pregnancies, and 1.8 vaginal deliveries. Two patients were lost to follow-up and excluded. Three complete radiofrequency treatments were performed in 31/50 patients, whereas 19 patients received 1–2 treatments. There were no adverse events from the radiofrequency treatment. No changes were found in resting pelvic muscle tone after Votiva treatment [Wilks’ lambda = 0.98, *F* (1, 45) = 0.86, *P* = 0.36]. The quantity of treatments seemed to impact mean values of maximal pelvic floor contraction [*F* (1, 45) = 105.14, *P* < 0.001]. On the patient questionnaire, patients felt subjective improvement correlated to number of treatments.

**Conclusions:**

Radiofrequency is safe for the treatment of pelvic floor dysfunction. This study showed no changes in resting pelvic muscle tone but an improvement in maximal pelvic floor contraction. A prospective randomized study is being conducted to further evaluate the efficacy of this technology.

## Abstract 2

Supplemental Digital Content is available in the text.

## INTRODUCTION

The American Society of Plastic Surgeons estimated a 39% increase in plastic surgeons performing vulvovaginal restoration procedures (surgical and nonsurgical) in the United States from 2015 to 2016.^1^ Nonsurgical vulvovaginal therapy has been one of the fastest growing areas in plastic surgery and urogynecology over the past 10 years.^2^ The first energy-based vulvovaginal rejuvenation device became available in Europe in 2008. By 2016, there were an estimated >500,000 procedures performed annually.^3,4^

Pelvic floor diseases (PFDs) are estimated to impact 24% of women in the United States (15% urinary incontinence, 3% pelvic organ prolapse, and 9% fecal incontinence).^5^ The prevalence of these conditions increases significantly with age,^5,6^ with a lifetime risk of undergoing a single operation for prolapse or incontinence of 11% and a reoperation rate of 30%.^5–8^ The aging population^6^ and rise of obesity^9–11^ have led to increased prevalence of PFD and increased rates of surgical procedures.^12^ However, studies indicate that providers are unsure of therapeutic options and are inadequately trained to manage these problems.^13^ Yet, PFD is a major source of morbidity and a burden on the healthcare system, with an estimated cost of US$83 billion by 2020.^14^ Current treatment options for PFD are limited and include biofeedback, laser, electrical muscle stimulation, and in certain cases, operative intervention.^5–7,15–19^

The increasing interest in pelvic floor restoration (PFR) is a reflection of decreased stigmatization of female health issues^2,6,7,19–21^ and demonstrated safety and efficacy of energy-based devices.^2,15,18,22–26^ Despite this, there are barriers preventing sound scientific evaluation of these devices including: lack of objective outcome measures, use of unvalidated surveys, paucity of case/control studies, and inadequate follow-up.

A number of energy-based devices, including radiofrequency (RF) and laser (CO_2_, Er:YAG) have been used to improve external genital appearance, vaginal laxity, and stress incontinence.^2,13,18,21–24^ Patients and clinicians often view these nonsurgical options as more attractive to invasive surgical treatment—with less downtime, discomfort, and cost. RF treatment may provide particular benefit in cases of disturbance to the genito-pelvic floor, where stretching of the vaginal introitus^13^ can lead to decreased sexual function, lubrication, genito-pelvic sensation, stress urinary incontinence, bowel incontinence, chronic pelvic pain, and pelvic organ prolapse.^13,15,16,23,27^

RF applied to the vaginal wall has been shown to stimulate proliferation of glycogen-enriched epithelium, neovascularization, and collagen formation^21^ by creating heat via impedance, as an electric current is conducted through the target tissues.^25,28^ Once these devices generate temperatures between 40°C and 45°C, an inflammatory cascade is initiated and heat shock proteins induce fibroblasts, which leads to neocollagenesis and elastogenesis.^2,21,22^ By controlling temperature at this level, new cells generate rather than forming scar tissue.

However, once dermal tissues reach temperatures >50°C there is a risk of thermal injury.^21^

This study describes the use of the Votiva bipolar RF device (InMode, Lake Forest, Calif.) for PFR in 50 patients experiencing PFD symptoms after vaginal childbirth. A transcutaneous electromagnetic muscle and nerve stimulator (UROstym, Mississauga, Ont.) commonly used in urogynecology was used to measure resting (UROstym min) and maximal (UROstym max) contraction of the pelvic floor pre- and post-RF treatments. The UROstym functions as a separate muscle stimulator and measuring device, uniquely providing objective data to evaluate the impact of the RF treatment. Further, this data provide insight to assess the potential effect of the RF device on muscle biofeedback, which is currently a standard treatment for PFR. Also, a patient symptom improvement index (PSI) was obtained to evaluate the patient’s perceived impact of the treatment.

## METHODS

A retrospective evaluation was conducted between April 2017 and May 2018 of consecutive patients undergoing vaginal RF treatment with Votiva. Inclusion criteria were patients at least 6 weeks postvaginal delivery with aforementioned symptoms of pelvic floor dysfunction. Clinical examination included either visually noting an open introitus, digital examination measuring the strength of patient’s maximal contraction, or visually noting the length of the genital hiatus (from urethra to fourchette; >5 cm was considered attenuated). Patients with active infections, unhealed lacerations, smokers, and those lost to follow-up were excluded from the study. Transcutaneous muscle and nerve stimulator (UROstym) was used pre- and post-RF treatments to measure resting tone and maximal contraction strength (mV). This biofeedback system includes rectal and vaginal probes that stimulate muscles and nerves in the pelvic floor. UROstym was used as a muscle contraction measuring device in addition to biofeedback treatment device (see **video, Supplemental Digital Content 1**, which demonstrates the Votiva PFR with the use of UROstym electrostimulation unit. This video is available in the “Related Videos” section of the Full-Text article on PRSGlobalOpen.com or available at http://links.lww.com/PRSGO/B39).

**Video Graphic 1.**
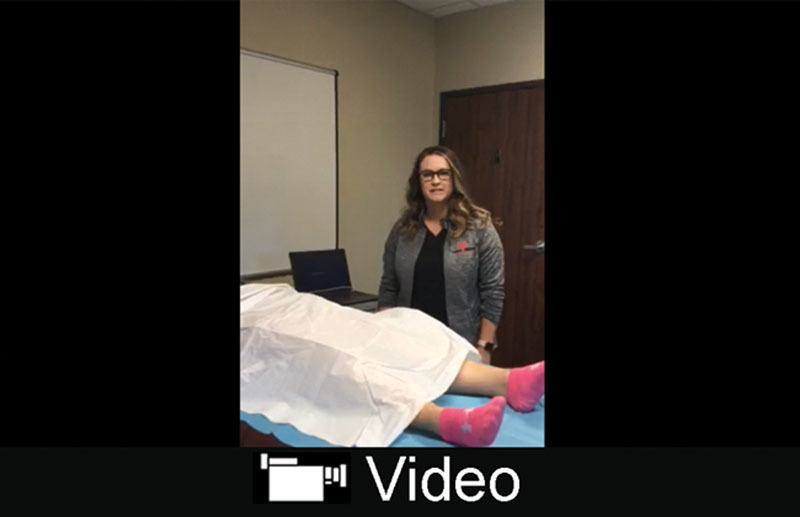
See video, Supplemental Digital Content 1, which demonstrates the Votiva PFR with the use of UROstym electrostimulation unit. This video is available in the “Related Videos” section of the Full-Text article on PRSGlobalOpen.com or available at http://links.lww.com/PRSGO/B39.

Treatments began 6 weeks postpartum as all lacerations/episiotomy sites had healed and patients returned to baseline hormone levels and sexual activity. Measurements were done before the first and second RF treatments and 2 weeks thereafter. PSI data were obtained, which included verbal symptom–based questions and sexual function assessment. The score also included the clinician’s ability to visualize a change in laxity. All patients received 3 UROstym measurements regardless of the number of RF treatments.

Data points collected included patient demographics (age, body mass index, race, medical history), number of pregnancies/vaginal deliveries, history of rectal tears, episiotomies, and vaginal laxity on examination by gynecologist (H.R.) (scale 0–4). The number of vaginal RF treatments and associated pre- and post-UROstym measurements were recorded in addition to detailed parameters of the RF treatment (intervals, duration, average internal/external time, and energy used). Any minor or major adverse events were recorded. Primary aims of the study were to identify safety, tolerability, and clinical efficacy of RF for PFR.

## RESULTS

A total of 50 women were included in the study with an average age of 32 (29–40) years old, average of 2.6 pregnancies (STD = 1.2), and 1.8 vaginal deliveries (STD = 1.2). Two patients were excluded after the first treatment as they had been lost to follow-up. Postpartum genitorectal trauma in this cohort included 5/50 (10%) with episiotomies, 4/50 (8%) with vaginal tears, and no reported rectal tears. Three complete RF PFR treatments were performed in 31/50 patients, whereas 19 patients received 1–2 treatments. Average time between treatments was 1.6 weeks (STD = 0.8) and average time of internal treatment was 9.4 minutes (STD = 1.0). There were no reported adverse events from the RF treatment. Patients were followed for 1 year from initial treatment.

To assess the RF treatment effects on resting muscle potential (UROstym min) and maximal muscle contraction (UROstym max), analysis of variance (ANOVA) test was employed. All patients were measured 3 times using the UROstym, regardless of the number of RF treatments completed. This allowed for us to evaluate if those who did not engage in all 3 treatments would have different values across time compared with those who engaged in the complete program. For statistical evaluation, we divided the cohort in 2 groups: a first group who completed all 3 treatments (31/50) and a second group who completed 1–2 treatments (19/50).

We hypothesized that certain factors may influence the UROstym min and UROstym max recordings, such as patient age and number of pregnancies. Number of pregnancies was not normally distributed; thus, this factor was dichotomized, with 1–2 pregnancies (N = 29) and ≥3 pregnancies (N = 21). Initial UROstym min and max served as control variables in the analyses.

### Impact of RF PFR on Resting Pelvic Muscle Tone (UROstym Min)

Focusing first on RF PFR on resting pelvic muscle tone (UROstym min), there seemed to be no statistically significant interaction effect of time to treatment (Wilks’ lambda = 0.98, *F* (1, 45) = 0.86, *P* = 0.36) or control variables (age, number of pregnancies) (*F* (1, 45) = 0.63, *P* = 0.430). The interaction effect between time and number of treatments was also not significant (Wilks’ lambda = 1.00, *F* (1, 45) = 0.40, *P* = 0.53). However, the time 1 control variable did exhibit a statistically significant main effect (*F*, (1, 45) = 35.75, *P* < 0.001), indicating that the time 1 value positively predicts subsequent values of UROstym min, which, intuitively, is not very meaningful. When analyzing the pairwise comparison between time 2 and time 3, there was no difference in mean values, indicating there was no effect of the treatment across time. The between-subjects interaction effect between time 1 and number of treatments was also not significant.

**Fig. 1.**
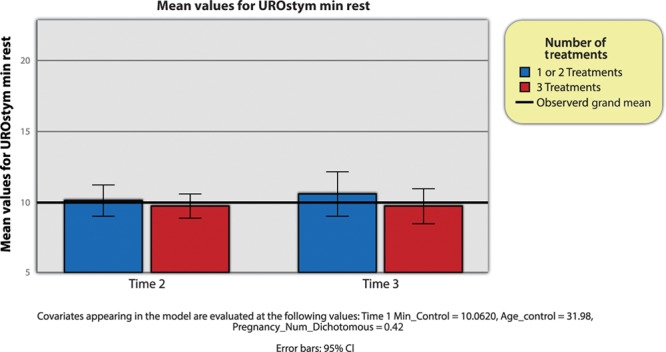
UROstym min across time and by group.

In other words, patients with lower resting pelvic muscle tone (UROstym min) at time 1 also had lower resting pelvic muscle tone (UROstym min) at time 2 and time 3. Thus, neither the passage of time nor the quantity of treatments seemed to impact the mean values of resting pelvic muscle tone (UROstym min) (Fig. 1).

### Impact of RF PFR on Maximal Pelvic Muscle Contraction (UROstym Max)

Similarly, when analyzing maximal pelvic floor contraction (UROstym max), there was no significant effect of time of measurement (Wilks’ lambda = 1.00, *F* (1, 45) = 0.12, *P* = 0.74), between-subjects effect (*F* (1, 45) = 3.12, *P* = 0.08) (though nearing significance), or control variables (age, pregnancies). Also, the interaction effect between time and number of treatments was not significant (Wilks’ lambda = 1.00, *F* (1, 45) = 0.01, *P* = 0.95).

However, the time 1 control variable did exhibit a statistically significant main effect (*F* (1, 45) = 105.14, *P* < 0.001), indicating that the time 1 value positively predicted subsequent values of maximal pelvic muscle contraction (UROstym max). The subsequent comparison between time 2 and time 3 was also statistically significant, which is indicative of a main effect for the passage of time by itself. The between-subjects interaction effect between time 1 and number of treatments was not significant. In other words, patients with higher UROstym max at time 1 will also have higher max at time 2 and time 3.

**Fig. 2.**
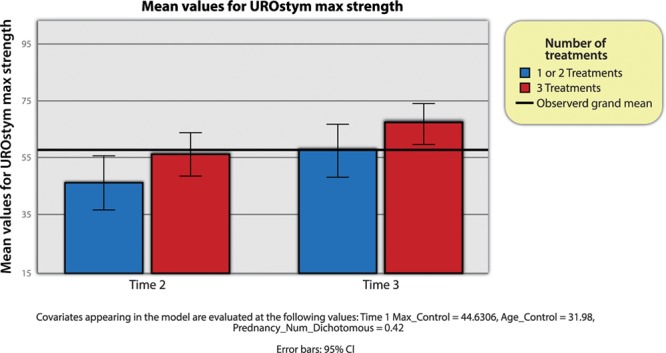
UROstym max across time and by group.

In sum, the quantity of treatments seemed to impact the mean values of UROstym max (Fig. 2). However, given a between-subjects main effect of time 1 on subsequent times, that the time 2 and 3 by time 1 interaction was nearing statistical significance, and that the sample size was relatively modest, more testing is warranted for this set of variables.

**Fig. 3.**
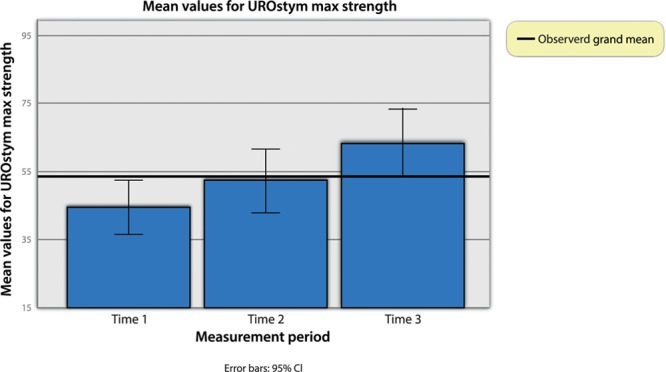
Pairwise comparisons of UROstym max at different times.

Based on the test results for UROstym max, it may be that too many independent /control variables are obscuring the relationship. Therefore, a simple repeated measures ANOVA with time 1, time 2, and time 3 values of UROstym max was computed. First, to ensure the assumption against sphericity was not violated, Mauchly’s test of sphericity was computed (*X*^2^ (2) = 4.01, *P* = 0.14). The ANOVA was found to be statistically significant (Wilks’ lambda = 0.48, *F* (2, 49) = 24.89, *P* < 0.001, eta-squared = 0.34). According to the pairwise comparisons, all mean values differed from each other (µ Time 1 = 44.63, SD Time 1 = 3.92; µ Time 2 = 52.39, SD Time 2 = 4.66; µ Time 3 = 63.48, SD Time 3 = 5.03) (Fig. 3).

**Fig. 4.**
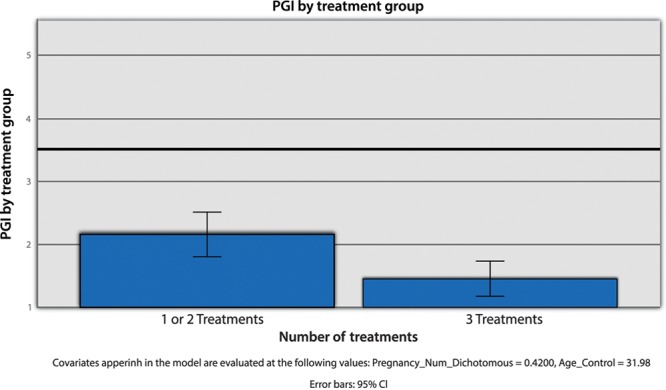
UROstym max strength across time.

In summary, when excluding the between-subjects independent variable and the control variables, there is a clear and significant relationship between treatments and measurement of maximal pelvic floor contraction (UROstym max) across time. That is, after each treatment, UROstym max increased a statistically significant amount (Fig. 4).

**Fig. 5.**
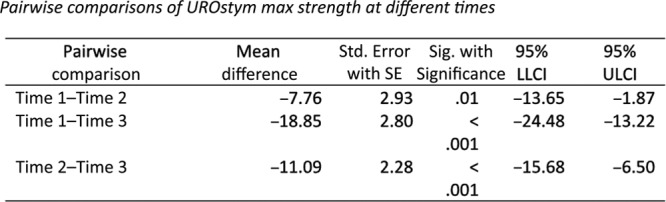
PSI treatment group. LLCI indicates lower limit CI; ULCI, upper limit CI.

The final element of this study was evaluation of the patient perception of improvement using a PSI with 5-level measure (0–4). The outcome of this test was more conclusive. A one-way ANOVA, with the dichotomous number of treatments variable serving as the independent factor and questionnaire score as the dependent variable, was conducted. Moreover, age and number of pregnancies served as control factors. The main effect of quantity of treatments was found to be statistically significant (*F* (1, 46) = 9.22, *P* = 0.004, eta-squared = 0.17). This indicated that those engaged in all 3 treatments exhibited values that were consistent with higher levels of progress including improved sexual function, lubrication, and decreased incontinence (µ = 1.45, SD = 0.68), whereas those who experienced fewer treatments exhibited values that were consistent with lower levels of progress (µ = 2.16, SD = 0.90). The effect size was large (Cohen’s d = 0.89) (Fig. 5).

## DISCUSSION

Animal models have been useful to understand histologic changes in nonsurgical PFR, yet there are limited clinical studies on RF treatments.^2,13^ One major barrier to investigation is the lack of a standardized measuring device for pelvic floor muscle strength and vaginal introital laxity.^2^ Several potential measuring devices have been used, including caliper measurement, balloon devices (such as barostat for measuring esophageal strictures), and comparative photographs—but all have been deemed inadequate or unreliable.^2,13^ Of the clinical trials that exist, most focus on vulvovaginal rejuvenation with favorable outcomes, demonstrating improvements in vaginal laxity, lubrication, arousal, without significant adverse events (ulceration, necrosis, scarring) or pain.^2,13,18,20–24,26^ Majority of patients report good tolerability of in-office procedures with a commonly reported feeling of warmth. There were no complications or adverse reactions. To our knowledge, no study has successfully used an objective measuring device to quantify pelvic floor function pre-and post-RF PFR treatment.

Our study findings significantly showed that RF improved maximal pelvic muscle contraction (UROstym max) measurements after the first treatment. Even when removing all control variables and only analyzing all 3 time points, the UROstym max increased at each time point. This indicates that UROstym max was positivity impacted by the RF treatment. Furthermore, this was the case for patients who only had 1–2 treatments. One possible explanation is that the first treatment was effective in starting a “tightening cascade” that made subsequent treatments less impactful. This may be explained by motor unit recruitment, which is a known phenomenon used to explain activation of additional motor units for increased contractile strength in a muscle.^29^ Also, it is possible that the known tightening effect of RF energy restores the muscle length/tension relationship allowing for increased contractile efficacy as described in the Frank–Starling relationship. A similar phenomenon was shown subjectively by Alinsod^25,28^ who reported improvements in stress incontinence, atrophic vaginitis, and orgasmic dysfunction most profoundly after the first RF treatment, with some additional improvement noted after the second and third treatments. Millheiser et al.^23^ similarly demonstrated greatest improvement within 1 month after RF treatment. This is also consistent with animal models that demonstrate stromal remodeling with fibroblast activation between 1 week and 1 month after treatment and variably increased muscularis collagen over 6-month posttreatment period. Future studies will clarify the impact of additional RF treatments beyond the initial one.

For pelvic floor resting potential (UROstym min), there seemed to be no change from time 2 to time 3 when controlling for time 1. Also, the number of RF treatments did not seem to impact resting potentials. However, this may be due to a relatively small sample size that was not sufficient to detect a change in this field. Subjectively, patients did report a decrease in resting muscle spasms which may indicate an improvement in resting muscle tone. Indeed, if the effect of UROstym min across time and groups is small, the sample size of the current study would be insufficient to detect the relationship. A post hoc power analysis was conducted, showing that a total sample size of 96 would be needed to detect a small effect—roughly double of what was used in the present study.

Finally, the PSI results indicated that patients who experienced all 3 RF treatments had significantly better assessments compared with those who only had 1 or 2 treatments. Patients stated that improvement was noticeable in areas of sexual function, lubrication, and urinary continence.

We identify a number of limitations inherent in the retrospective nature of this study, including the potential for data inaccuracies and confines in study design. It would have been beneficial to have a control or sham arm to account for the potential placebo effect and case randomization (ie, complete treatment versus 1 versus 2 treatments). This would have made a stronger case for causality, as comparing groups post hoc introduces experimental bias. Another weakness of this study was that only 31/50 patients completed all 3 RF treatments. Although this was not the intended nature of the study, it did allow for us to assess the impact of 1 or 2 treatments versus 3 treatments.

Despite the retrospective nature of this analysis, we were able to discern a number of significant findings as previously shown. Moreover, this study is the first to our knowledge that uses an objective measure to evaluate effectiveness of RF for PFR.

## CONCLUSIONS

This study demonstrates the safety of using RF energy for PFR after vaginal delivery. Our data found no adverse events in 50 consecutive patients. A significant correlation was found between treatment and maximal contraction of pelvic floor muscles using the UROstym device. Furthermore, we suggest a “tightening cascade” phenomenon, where the impact of 1 treatment was all that needed to initiate patient improvement. Also, the PSI used correlated to subjective improvement with each treatment.

RF PFR may potentially fill a treatment gap of pelvic floor disorders. A powered prospective randomized double-blinded study is needed to further clarify the role of this technology.

## References

[1] ASoP Surgeons. Plastic Surgery Statistics Report 2016. 2016 Available at: https://www.plasticsurgery.org/documents/News/Statistics/2016/plastic-surgery-statistics-full-report-2016.pdf. Accessed 3 September 2018.

[2] Qureshi AA Tenenbaum MM Myckatyn TM Nonsurgical vulvovaginal rejuvenation with radiofrequency and laser devices: a literature review and comprehensive update for aesthetic surgeons. Aesthet Surg J. 2018;38:302 311. 29040373 10.1093/asj/sjx138

[3] Moretti M Feminine Rejuvenation Market Growth Surges via Energy-Based Devices. 2018 Available at: http://www.aestheticchannel.com/cosmetic-surgery/feminine-rejuvenation-market-growth-surges-energy-based-devices/page/0/2. Accessed 27 August 2018.

[4] Cosmetic Surgery National Data Bank Statistics. Aesthet Surg J. 2017;37(suppl 2):1 29. 10.1093/asj/sjx076 28388734

[5] Memon HU Handa VL Vaginal childbirth and pelvic floor disorders. Womens Health (Lond). 2013;9:265 277; quiz 276. 23638782 10.2217/whe.13.17 PMC3877300

[6] Nygaard I Barber MD Burgio KL; Pelvic Floor Disorders Network. Prevalence of symptomatic pelvic floor disorders in US women. JAMA. 2008;300:1311 1316. 18799443 10.1001/jama.300.11.1311 PMC2918416

[7] Olsen AL Smith VJ Bergstrom JO Epidemiology of surgically managed pelvic organ prolapse and urinary incontinence. Obstet Gynecol. 1997;89:501 506. 9083302 10.1016/S0029-7844(97)00058-6

[8] Shamliyan T Wyman J Bliss DZ Prevention of urinary and fecal incontinence in adults. Evid Rep Technol Assess (Full Rep). 2007(161):1 379. PMC4781595 18457475

[9] Hendrix SL Clark A Nygaard I Pelvic organ prolapse in the Women’s Health Initiative: gravity and gravidity. Am J Obstet Gynecol. 2002;186:1160 1166. 12066091 10.1067/mob.2002.123819

[10] Ogden CL Carroll MD Fryar CD Prevalence of obesity among adults and youth: United States, 2011-2014. NCHS Data Brief. 2015(219):1 8. 26633046

[11] Swift S Woodman P O’Boyle A Pelvic Organ Support Study (POSST): the distribution, clinical definition, and epidemiologic condition of pelvic organ support defects. Am J Obstet Gynecol. 2005;192:795 806. 15746674 10.1016/j.ajog.2004.10.602

[12] Erekson EA Lopes VV Raker CA Ambulatory procedures for female pelvic floor disorders in the United States. Am J Obstet Gynecol. 2010;203:497.e1 497.e5. 20739015 10.1016/j.ajog.2010.06.055 PMC2975837

[13] Sekiguchi Y Utsugisawa Y Azekosi Y Laxity of the vaginal introitus after childbirth: nonsurgical outpatient procedure for vaginal tissue restoration and improved sexual satisfaction using low-energy radiofrequency thermal therapy. J Womens Health (Larchmt). 2013;22:775 781. 23952177 10.1089/jwh.2012.4123

[14] Bahrampour T The Hidden Medical Epidemic Few Women Have Been Willing To Talk About, Until Now. 2015 Accessed 27 August 2018.

[15] Barrett G Pendry E Peacock J Women’s sexual health after childbirth. BJOG. 2000;107:186 195. 10688502 10.1111/j.1471-0528.2000.tb11689.x

[16] Griffiths A Watermeyer S Sidhu K Female genital tract morbidity and sexual function following vaginal delivery or lower segment caesarean section. J Obstet Gynaecol. 2006;26:645 649. 17071432 10.1080/01443610600903701

[17] Handa VL Blomquist JL Knoepp LR Pelvic floor disorders 5-10 years after vaginal or cesarean childbirth. Obstet Gynecol. 2011;118:777 784. 21897313 10.1097/AOG.0b013e3182267f2f PMC3178744

[18] Krychman ML Vaginal laxity issues, answers and implications for female sexual function. J Sex Med. 2016;13:1445 1447. 27567072 10.1016/j.jsxm.2016.07.016

[19] Perone N Pelvic floor disorders 5-10 years after vaginal or cesarean childbirth. Obstet Gynecol. 2012;119:182; author reply 182. 10.1097/AOG.0b013e31823f0c82 22183228

[20] Magon N Alinsod R Female cosmetic genital surgery: delivering what women want. J Obstet Gynaecol India. 2017;67:15 19. 28242962 10.1007/s13224-016-0930-y PMC5306104

[21] Tadir Y Gaspar A Lev-Sagie A Light and energy based therapeutics for genitourinary syndrome of menopause: consensus and controversies. Lasers Surg Med. 2017;49:137 159. 28220946 10.1002/lsm.22637 PMC5819602

[22] Vos JA Livengood RH Jessop M Non-ablative hyperthermic mesenchymal regeneration: a proposed mechanism of action based on the Viveve model. Proc SPIE. 2011:7901:7901041 7901048.

[23] Millheiser LS Pauls RN Herbst SJ Radiofrequency treatment of vaginal laxity after vaginal delivery: nonsurgical vaginal tightening. J Sex Med. 2010;7:3088 3095. 20584127 10.1111/j.1743-6109.2010.01910.x

[24] Magon N Alinsod R ThermiVa: the revolutionary technology for vulvovaginal rejuvenation and noninvasive management of female SUI. J Obstet Gynaecol India. 2016;66:300 302. 27382227 10.1007/s13224-016-0868-0 PMC4912496

[25] Alinsod RM Transcutaneous temperature controlled radiofrequency for orgasmic dysfunction. Lasers Surg Med. 2016;48:641 645. 27197701 10.1002/lsm.22537 PMC5084776

[26] Nappi RE Palacios S Panay N Vulvar and vaginal atrophy in four European countries: evidence from the European REVIVE Survey. Climacteric. 2016;19:188 197. 26581580 10.3109/13697137.2015.1107039 PMC4819825

[27] Yang SH Yang JM Wang KH Biologic correlates of sexual function in women with stress urinary incontinence. J Sex Med. 2008;5:2871 2879. 18778309 10.1111/j.1743-6109.2008.00985.x

[28] Alinsod RM Transcutaneous temperature controlled radiofrequency for atrophic vaginitis and dyspareunia. J Minim Invasive Gynecol. 2015;22(suppl):S226 S227. 10.1016/j.jmig.2015.08.798 27679110

[29] Heckman CJ Enoka RM Motor unit. Compr Physiol. 2012;2:2629 2682. 23720261 10.1002/cphy.c100087

